# Bystander effect of SARS-CoV-2 spike protein on human monocytic THP-1 cell activation and initiation of prothrombogenic stimulus representing severe COVID-19

**DOI:** 10.1186/s12950-022-00325-8

**Published:** 2022-12-30

**Authors:** Tapas Patra, Ranjit Ray

**Affiliations:** 1 Departments of Internal Medicine, Division of Infectious Diseases, Allergy & Immunology, Edward A. Doisy Research Center, 1100 South Grand Blvd, MO 63104 Saint Louis, USA; 2 Molecular Microbiology & Immunology, Saint Louis University, 63104 Saint Louis, Missouri, MO USA

**Keywords:** COVID-19, Inflammation, Monocyte, Complement, Platelet, Cytokine

## Abstract

**Background:**

Hypercoagulable state and thromboembolic complications are potential life-threatening events in COVID-19 patients. Our previous studies demonstrated that SARS-CoV-2 infection as well as viral spike protein expressed epithelial cells exhibit senescence with the release of inflammatory molecules, including alarmins.

**Findings:**

We observed extracellular alarmins present in the culture media of SARS-CoV-2 spike expressing cells activate human THP-1 monocytes to secrete pro-inflammatory cytokines to a significant level. The release of THP-1 derived pro-inflammatory cytokine signature correlated with the serum of acute COVID-19 patient, but not in post-COVID-19 state. Our study suggested that the alarmins secreted by spike expressing cells, initiated phagocytosis property of THP-1 cells. The phagocytic monocytes secreted complement component C5a and generated an autocrine signal via C5aR1 receptor. The C5a-C5aR1 signal induced formation of monocyte mediated extracellular trap resulted in the generation of a prothrombogenic stimulus with activating platelets and increased tissue factor activity. We also observed an enhanced C5a level, platelet activating factor, and high tissue factor activity in the serum of acute COVID-19 patients, but not in recovered patients.

**Conclusion:**

Our present study demonstrated that SARS-CoV-2 spike protein modulates monocyte responses in a paracrine manner for prothrombogenic stimulus by the generation of C5a complement component.

## Background

SARS-CoV-2 is the etiological agent for Coronavirus associated COVID-19. A large number of confirmed COVID-19 cases and deaths have already been reported. The pathological consequences of COVID-19 are under intense investigation for the use of precise therapeutic modalities [1].

Coagulopathy and microvascular complications are often associated with COVID-19 related mortality. Thrombocytopenia and increased D-dimer level have demonstrated as early predictors of critically ill COVID-19 patients [2]. Complement activation associates with COVID-19 inflammatory responses and complement can fuel thrombo-inflammation [3, 4]. Although SARS-CoV-2 infection restricts particularly in pulmonary region, but systemic hyper-inflammatory condition pretends thrombo-inflammatory state that results various tissue damage and multiorgan failure [5–8]. Circulating monocytes are the most effector immune cells respond to innate immune response against pathogen and play as a catalyst of systemic hyper-inflammatory condition [9].

In our previous report, we hypothesized that SARS-CoV-2 infection of pneumocytes potentiates trans-signaling to the other cells that promotes pathogenesis [10]. Understanding pathophysiology of COVID-19 has so far focused on epithelial, endothelial cells and lymphocytes. A relatively less attention was attributed to the circulatory monocytes and their effector mechanisms for inflammatory state of SARS-CoV-2 infection. COVID-19 severity associated with infiltrating inflammatory monocytes exhibited in a hyperactivated state [11, 12]. In vitro studies showed SARS-CoV-2 protein expressing epithelial cells display release of increased soluble mediators to activate innate sensors in monocytes [13, 14]. Here, we have investigated the thrombo-inflammatory response of activated human THP-1 monocytes in response to the bystander effect of SARS-CoV-2 spike expressing cells and correlated with the inflammatory response attributing to COVID-19 state of patients.

### Materials and methods

#### Patient samples

Coded serum samples from six hospitalized patients with acute SARS-CoV-2 infection, six patients recovered from SARS-CoV-2 infection with virus negative and four from uninfected healthy volunteers were obtained from Saint Louis University Hospital COVID-19 repository (Institutional Review Board Approval Numbers 26,646 and 27,790). Consents for research use of sera were obtained from the patients and volunteers [15].

#### Cell culture and transient transfection

Human lung epithelial cell line A549 was cultured in DMEM (Hyclone) containing 10% FBS, and 1% penicillin-streptomycin (Sigma). The cells were maintained in a humidified atmosphere at 37˚C with 5% CO_2_. For transient transfection, cells were sub-cultured in a 6-well plate to 60% confluence and transfected with plasmid DNA (pcDNA3.1-SARS-Cov-2-Spike MC-0101087-5834, BEI Resources) or empty vector construct (2.5 mg/well) using Lipofectamine 3000 (Life Technologies) following the manufacturer’s instruction. Culture supernatant (CM) was collected passing through 0.22 µM filter after 72 h. Human monocytic cell line THP-1 was cultured in RPMI containing 10% CFS (Gibco), 1% penicillin-streptomycin (Sigma), 1% L-glutamine (Hyclone), 25 mM HEPES (Sigma) and 12.5 nM 2-ME (Sigma) to analyze hyperinflammatory response.

#### Inhibitors and inflammatory molecules treatment

THP-1 cell line was treated with different inhibitors- 2 µM of ST2825 (MedChemExpress), 5 µM of Pepstatin A (Sigma), 50 nM of PMX205 (Tocris), 5 µM of YCG063 (Calbiochem); and different human recombinant inflammatory molecules − 0.01 ng/ml of IL-6, 0.01 ng/ml of TNF-α (Sigma), 10 ng/ml of IL-1α (ThermoFisher), 100 ng/ml of HMGB1, 0.05 ng/ml of C5a (R&D system), respectively.

#### ELISA

Patient serum samples and cell culture supernatants of THP-1 cells exposed of the CM of spike expressing cells collected at second day were analyzed for the concentration of IL-6, TNF-α, IL-1α (Sigma), IL-1β, IL-8, IL-10, IL-4, IL-13, TGF-β1 (Invitrogen) and HMGB1 (Novus) using ELISA kits following the manufacturer’s instructions. We also analyzed level of C5a (Invitrogen), platelet activation factor (Lifespan), intracellular ROS (Cayman) by ELISA following supplier’s guidelines from patient sera and cell culture supernatant of THP-1 cells exposed to the CM of spike expressing cells collected at different days.

#### Phagocytosis assay

Phagocytic activity of monocytes was assessed at different time intervals by fluorometric measurement using Vybrant™ phagocytosis assay kit (Invitrogen) following the manufacturer’s instructions. Monocytes in culture were pre-incubated with fluorescein-labeled *E. coli* bioparticle at 37 °C for 1 h and removed the bioparticle for wash. Fluorescence was measured from bioparticle using an fluorometric plate reader (TECAN) with 480 nm excitation and 520 nm emission.

#### Western blot

Western blot analysis was performed by Cell lysates prepared from THP-1 cells following exposure to culture media (CM) of spike expressing cells and treated with different inhibitors or inflammatory molecules for two or four days following our previous study [16]. Commercially available antibodies phospho-MyD88 [Tyr257] (ThermoFisher), phospho-ERK [Thr202/Tyr204], phospho-PKC [Ser660] (CellSignaling), C5aR1/CD88, Cryopyrin (SantaCruz) were used for western blot analysis.

#### qRT-PCR

THP-1 cells exposed CM of spike expressing cells and treated with different inhibitors or inflammatory molecules for four days were used for qRT-PCR assay following our previous study [17]. For detection, GAPDH (forward primer: 5′-CATGTTCGTCATGGGTGTGAACCA-3′; reverse primer: 5′-AGTGATGGCATGGACTGTGGTCAT-3′), TF (forward primer: 5′-ACTG GAATTCATATGGCTCTGGGCTCTTC-3′; reverse primer: 5′-TTCATATGTCAGGCACTAC AAATACTG-3′), C5aR1 (forward primer: 5′-GACCCCAGGAGACCAGAACATG-3′; reverse primer: 5′-TACATGTTGAGCAGGATGAGGGA-3′), and C5aR2 (forward primer: 5′-TGCTGT TTGTCTCTGCCCATC-3′; reverse primer: 5′-GTCAGCAGGATGATGGAGGG-3′) were used.

#### Flow cytometry

THP-1 cells were cultured in the presence of CM of spike expressing cells with or without ST2825 for two days, collected and stained with APC-conjugated anti-HLA-DR (BioLegend) antibody for 30 min and fixed. Surface expression of monocyte activation marker HLA-DR was analyzed using flow cytometry.

#### Immunofluorescence microscopy

THP-1 cells were grown in poly-L-lysine coated 8-well chamber slide and treated with CM of spike expressing cells with or without inhibitors and human recombinant C5a for five days. Cells were fixed with 3.7% formaldehyde, permeabilized using 0.2% Triton X-100, and blocked with 5% BSA in room temperature for 2 h. Cells were incubated with the primary antibody of myeloperoxidase (SantaCruz) and citrulline histone 3 (Novus) for overnight at 4 °C. After incubation, cells were stained with appropriate fluorescence-conjugated secondary antibody (Invitrogen) for 1 h at room temperature. Stained cells were visualized by immunofluorescence microscopy (Leica).

#### Tissue factor activity assay

Tissue factor activity was measured from patient serum samples and cell lysates of THP-1 pre-exposed to the CM of spike expressing cells treated with or without inhibitors collected on day two and five by using TF human chromogenic activity assay kit (Abcam) following supplier’s instruction. The presence of active tissue factor-FVIIa converted coagulation factor FXa as a substrate into active chromogenic product was measured from the absorbance at 405 nm on a spectrometric plate reader (TECAN).

#### Platelet activation assay

Blood samples from healthy donors were collected into vacutainers containing sodium citrate. Platelet-rich plasma was prepared by centrifuging whole blood at 100 g for 20 min. Platelets were washed by citrate dextrose buffer, centrifuging at 350 g for 20 min, and suspending in Tyrode’s buffer. Platelets were further incubated for one hour with fifth days activated monocytes. The platelets were collected from co-culture conditions and labeled with FITC-conjugated anti-CD62P and PE-conjugated anti-CD41 (BioLegend) for 30 min at room temperature. Platelets were fixed and analyzed by flow cytometry.

### Statistical analysis

GraphPad Prism 7 was used to analyze the experimental data. All experiments were performed at least three times for reproducibility. The results are presented as mean ± standard deviation. Non-parametric Mann-Whitney U test and paired two-tailed *t*-test analyses were performed to compare the mean values between the two groups. Statistical significance was considered as *p* < 0.05.

## Results

### Culture medium from SARS-CoV-2 spike transfected epithelial cells induces pro-inflammatory cytokine secretion from THP-1 monocytes

**Fig. 1:**
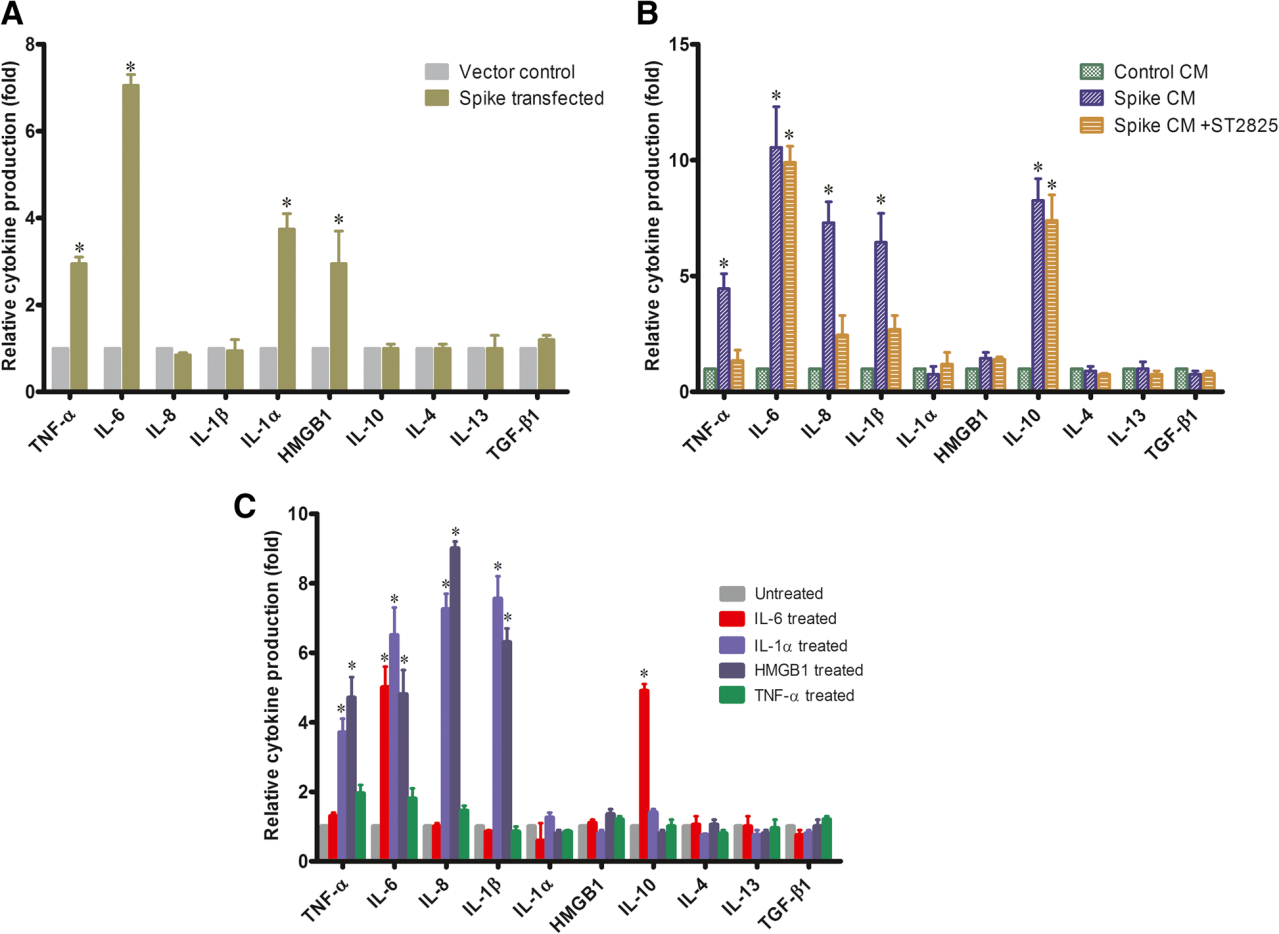
SARS-CoV-2 spike protein influences cytokine production. The relative level of pro-inflammatory cytokines TNF-α, IL-6, IL-8, IL-1β, IL-1α, HMGB1, and anti-inflammatory cytokines IL-10, IL-4, IL-13, TGF-β1 were measured by ELISA from the culture supernatant of (**A**) A549 cells transfected with SARS-CoV-2 spike gene construct or empty vector after two days of incubation. **B** THP-1 cells exposed to CM from spike expressing cells in the presence or absence of ST2825 for two days were analyzed for cytokine production. **C** THP-1 cells treated with TNF-α, IL-6, IL-1α, HMGB1 for two days. The results are presented as mean ± standard deviation and ‘*’ represents analyses of data only showing statistical significance *p* < 0.05

Our previous studies revealed that SARS-CoV-2 infected as well as SARS-CoV-2 spike transfected epithelial cells exhibit senescence and release inflammatory molecules exerting a paracrine effect on endothelial cells [15, 18]. Here, we analyzed the release of pro- and anti-inflammatory cytokines from the culture supernatants of SARS-CoV-2 spike transfected A549 pulmonary epithelial cells. Approximately 3.2-fold increase in TNF-α, 7.2-fold increase in IL-6, 4-fold increase in IL-1α, and 3-fold increase in HMGB1 secretion were noted in comparison to mock transfected A549 cells. The level of other cytokines, IL-8, IL-1β, IL-10, IL-4, IL-13, and TGF-β1, remained unchanged (Fig. 1A). We investigated whether the presence of these inflammatory molecules in SARS-CoV-2 spike expressing epithelial cell culture media can exert a bystander effect on surrounding monocytes to secrete different cytokines. For this, we incubated human monocytic cell line THP-1 with the conditioned culture medium collected from SARS-CoV-2 spike expressing A549 pulmonary epithelial cells (spike CM) for two days and analyzed extracellular cytokine levels. A significant increase in pro-inflammatory cytokines, TNF-α, IL-6, IL-8 IL-1β, and anti-inflammatory cytokine IL-10 were observed in the culture supernatant of SARS-CoV-2 spike transfected THP-1 cells (Fig. 1B). The levels of specific alarmin, IL-1α and HMGB1, previously available in the CM of spike expressing A549 cells were unchanged in the THP-1 culture supernatant cells treated either as control CM or spike CM. The other anti-inflammatory cytokines- IL4, IL-13 and TGF-β1, remained unaffected (Fig. 1B). A wide range of inflammatory molecule receptor, including tumor necrosis factor receptor (TNFR), IRAK, and TLRs (except TLR3) initiate signaling pathways through myeloid differentiation factor 88 (MyD88) activation [19]. We analyzed whether interruption of MyD88 activation modifies the cytokine secretion of spike CM exposed THP-1 cells. A specific MyD88 inhibitor ST2825 that prevent dimerization of MyD88 molecule was used and a reduced level of secreted pro-inflammatory cytokines TNF-α, IL-8 and IL-1β, except IL-6, was observed (Fig. 1B). Next, we analyzed to identify the inflammatory modulator present in spike CM to trigger the cytokine release from THP-1 cells. We found that exogenous treatment of alarmin IL-1α and HMGB1 induced secretion of pro-inflammatory cytokines (TNF-α, IL-6, IL-8 and IL-1β); whereas exogenous IL-6 treatment induced secretion of anti-inflammatory cytokine IL-10 and IL-6 (Fig. 1C).

### THP-1 derived cytokine signature correlates with COVID-19 patient serum

**Fig. 2:**
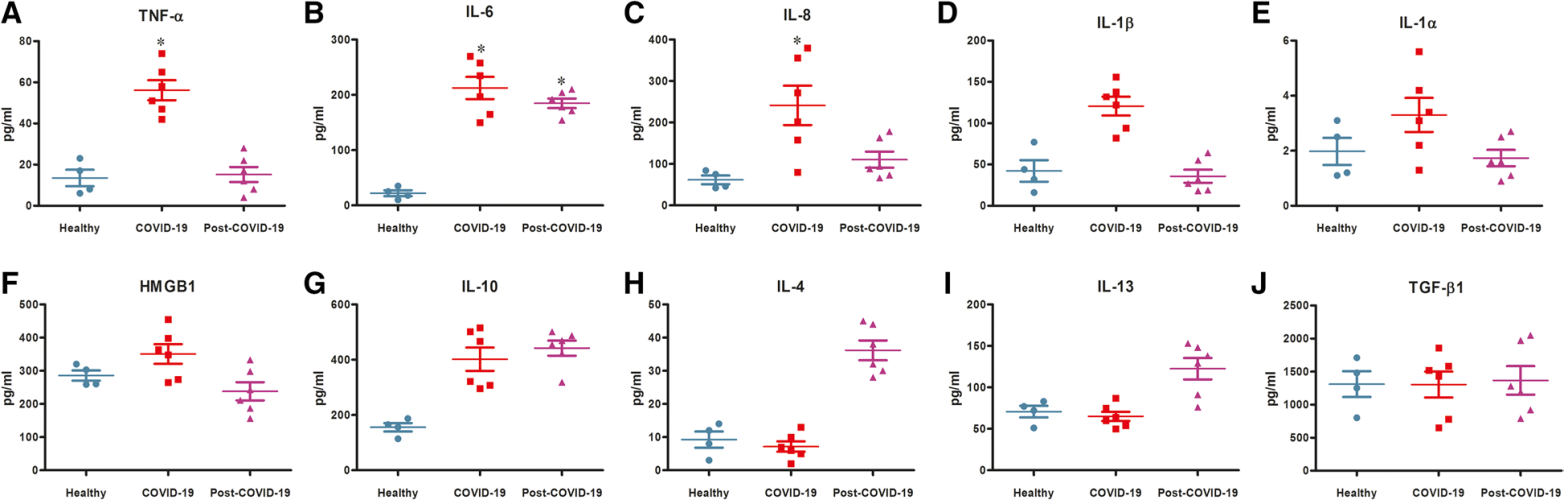
Difference in cytokine pattern in the acute and recovered COVID-19 patient sera. The status of (**A-F**) pro-inflammatory cytokines TNF-α, IL-6, IL-8, IL-1β, IL-1α, HMGB1, and (**G-J**) anti-inflammatory cytokines IL-10, IL-4, IL-13, TGF-β1 were measured by ELISA from the serum samples of uninfected healthy volunteers (*n* = 4), severe COVID-19 patients (*n* = 6) and recovered post-COVID-19 patients (*n* = 6). The results are presented as mean ± standard deviation and ‘*’ represents analyses of data only showing statistical significance *p* < 0.05

To correlate cytokine secretion pattern from THP-1 cells originally reflects on COVID-19 patients, we performed ELISA to determine the concentration of pro- and anti-inflammatory cytokines from the serum samples of uninfected healthy volunteers, hospitalized severe COVID-19 patients and recovered post-COVID-19 patients. The results showed that the level of pro-inflammatory cytokines, TNF-α, IL-6, IL-8, and IL-1β were significantly elevated in severe COVID-19 patients (Fig. 2 A-D), but the alarmin molecules IL-1α and HMGB1 were marginally higher (Fig. 2E and F). In the serum of severe COVID-19 patients, only anti-inflammatory cytokine IL-10 was significantly increased (Fig. 2G). The other anti-inflammatory cytokines IL-4 and IL-13 were raised only in recovered post-COVID-19 patients (Fig. 2 H and I), whereas TGF-β1 remained unchanged in all cases (Fig. 2 J). However, the elevated concentration of IL-6 and IL-10 persisted also in recovered post-COVID-19 sera (Fig. 2B and G). This information correlated with the signature of pro-inflammatory cytokines generated from our in vitro study and from the serum of severely ill COVID-19 patients.

### Alarmin release activates THP-1 cells expressing SARS-CoV-2 spike protein

**Fig. 3:**
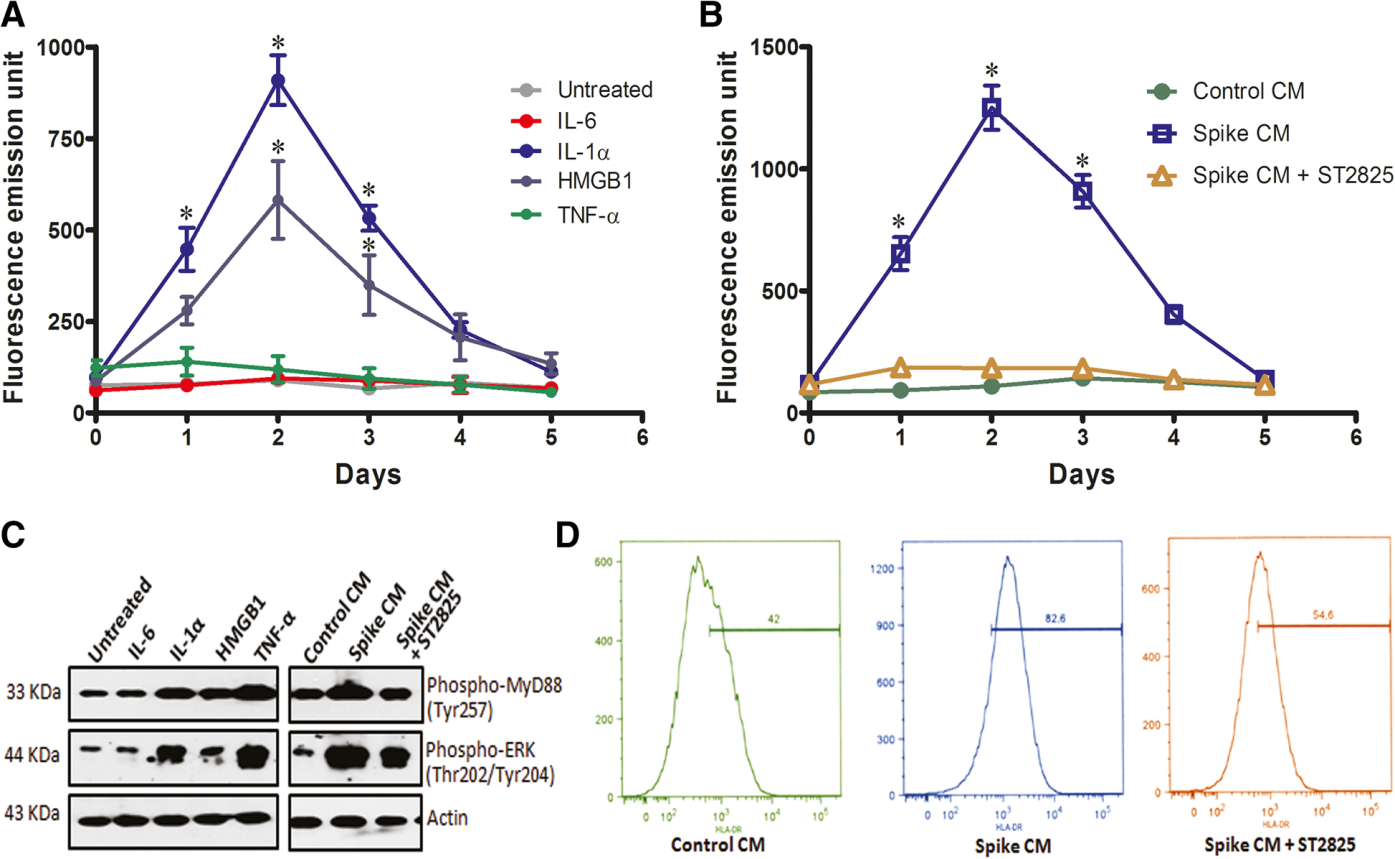
Activation of THP-1 monocytes exposed to the culture medium from SARS-CoV-2 spike expressing cells. Phagocytosis capacity of THP-1 cells was analyzed at different days by fluorometric assay from engulfed fluorescence tagged bioparticle (**A**) THP-1 cells were treated with IL-6, IL-1α, HMGB1, TNF-α; and (**B**) exposed to CM from spike expressing cells in the presence or absence of ST2825. **C** Phospho-MyD88 and phospho-ERK expression by Western blot analysis of THP-1 cells exposed to CM from spike expressing cells in the presence or absence of ST2825; and exogenous TNF-α, IL-6, IL-1α, and HMGB1 after incubation for two days. Expression level of actin in each lane from the same gel is shown for total protein load and comparison. **D** Monocyte activation was analyzed by flow cytometry for HLA-DR expression in THP-1 cells exposed to CM from virus spike expressing cells in the presence or absence of ST2825 for two days. The results are presented as mean ± standard deviation and ‘*’ represents analyses of data only showing statistical significance *p* < 0.05

Initialization of phagocytosis mechanism and release of pro-inflammatory cytokines and are the important features of activated monocyte related innate immune response [9]. Phagocytosis capacity was estimated at different time intervals by fluorometric assay for engulfing fluorescence tagged bioparticles of THP-1 cells treated with IL-6, IL-1α, HMGB1, or TNF-α. A maximum phagocytosis activity was observed with THP-1 cells after treatment with external IL-1α and HMGB1 for two days (Fig. 3A). Similar observation was also noticed in spike CM treated THP-1 cells (Fig. 3B) as anticipated for the presence of alarmin molecules (IL-1α and HMGB1) at a high level in the CM of SARS-CoV-2 spike expressing epithelial cells. Interestingly, introduction of MyD88 inhibitor ST2825 resulted in an inhibition of phagocytosis in spike CM exposed cells (Fig. 3B). Tyrosine phosphorylation of MyD88 adapts the inflammatory receptor mediated signaling through activation of extracellular signal-regulated kinase (ERK) to initiate innate immune responses, like phagocytosis, pro-inflammatory cytokine release [20, 21]. Our Western blot analysis showed higher phosphorylation at tyrosine-257 site of MyD88 and threonine-202/tyrosine-257 site of ERK molecule in THP-1 following IL-1α, HMGB1 and TNF-α treatment, or spike CM exposure (Fig. 3C). Enhanced HLA-DR expression on human monocyte cell surface acts as an early predictive marker for response to pathogens [22]. We also observed enhanced monocyte activation marker HLA-DR by flow cytometry on THP-1 cells exposed to the CM from spike expressing cells, and treatment with ST2825 reduced HLA-DR expression on cell surface (Fig. 3D). Elevated HLA-DR expression on monocytes were observed in mild to moderate COVID-19 patients [23]. These results indicated that SARS-CoV-2 spike expressing epithelial cells activate THP-1 monocytes to generate a pro-inflammatory response in a bystander manner.

### Activated THP-1 cells generate C5a complement component mediated autocrine signal

**Fig. 4:**
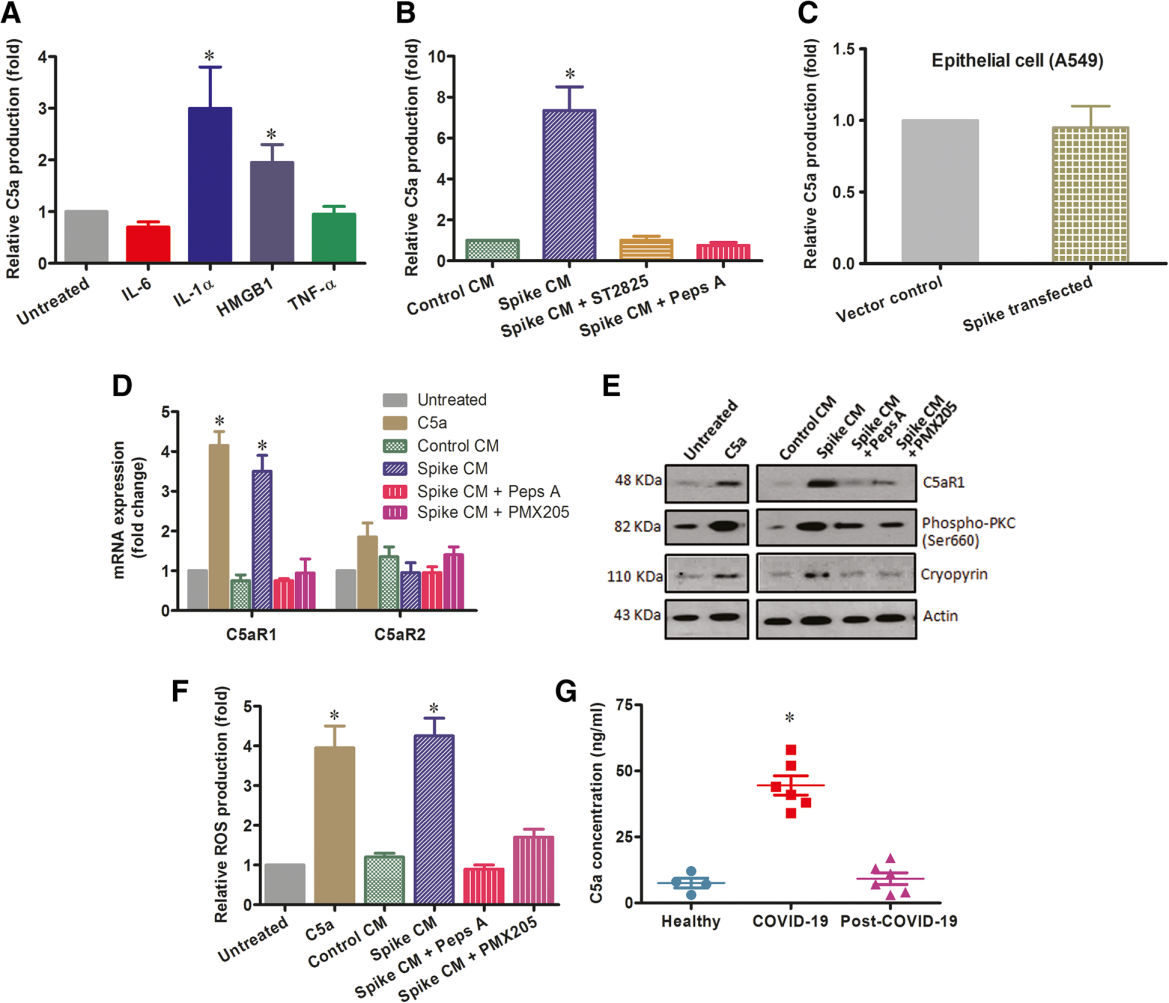
Activated THP-1 stimulates C5a-C5aR1 axis. The relative level of complement factor C5a was measured by ELISA from the culture supernatant of THP-1 cells (**A**) treated with IL-6, IL-1α, HMGB1, or TNF-α for four days; and (**B**) exposed to CM from spike expressing cells in the presence or absence of ST2825 or Peps A for four days. **C** Relative C5a level in the CM from A549 cells transfected with SARS-CoV-2 spike gene construct or empty vector is shown. **D** mRNA expression status of C5aR1 and C5aR2 analyzed by qRT-PCR from the total RNA of THP-1 cells exposed to the CM from SARS-CoV-2 spike expressing cells in the presence or absence of ST2825 or Peps A and exogenous C5a incubation for four days. **E** Expression of C5aR1, phospho-PKC, and cryopyrin were analyzed by Western blot from THP-1 cell lysates prepared after exposure to CM in the presence or absence of ST2825, Peps A, or exogenous C5a for four days. Expression level of actin in each lane from the same gel is shown as a total protein load for comparison. **F** Intracellular reactive oxygen spices were measured by fluorometric assay of THP-1 cells prior exposed to CM from virus spike expressing cells in the presence or absence of ST2825, Peps A, or exogenous C5a for four days. **G** The concentration of complement factor C5a was measured by ELISA from sera of uninfected healthy volunteers (*n* = 4), severe COVID-19 patients (*n* = 6) and post-COVID-19 patients (*n* = 6). The results are presented as mean ± standard deviation and ‘*’ represents analyses of data only showing statistical significance *p* < 0.05

Complement activation appears as a critical regulator of innate immune response. Generation of C5a from the cleavage of complement component C5 is an indication of complement activation process. Monocyte contains storage of C5 and C5a [24]. Complement factors are also produced locally by many immune cells, including monocytes. Recent studies showed sensing exogenous damage associated molecular patterns contribute phagocytosis by human monocytes and lead to C5a generation [25–27]. We already observed that alarmins release from SARS-CoV-2 spike expressing cells induce THP-1 phagocytosis. We were examining whether these phagocytic monocytes can generate C5a. We found that alarmin IL-1α or HMGB1 treatment induces C5a release as measured by ELISA from the culture supernatant of THP-1 cells on day four (Fig. 4 A). THP-1 cells secreted significant level of C5a after four days’ exposer with spike CM, but the C5a production was hampered in the presence of MyD88 inhibitor ST2825 and aspartic protease inhibitor Pepstatin A (Fig. 4B). To examine the involvement of phagocytosis in C5a generation, we used aspartic-protease inhibitor Pepstatin A (Peps A) since aspartic-protease is involved in phagolysosomal degradation process and may be responsible for the cleavage C5 to produce C5a. However, we did not observe a considerable level of C5a in the culture medium of SARS-CoV-2 expressing A549 cells (Fig. 4 C). Binding of C5a to its receptor C5aR1 or C5aR2 stimulates expression of these receptors [28]. We observed that mRNA expression of C5aR1 was upregulated in spike CM as well as exogenous C5a incubated THP-1 cells. Peps A and C5aR1 specific hexapeptide inhibitor PMX205 treatment led to suppress the upregulation, while the C5aR2 mRNA remained unchanged (Fig. 4D). C5aR1, as a GPCR family protein, after binding with C5a activates protein kinase C (PKC) and induces inflammasome related protein cryopyrin [28–30]. Western blot analysis showed elevated expression of C5aR1, phospho-PKC (serine 660) and cryopyrin in THP-1 following spike CM and exogenous C5a treatment. However, C5aR1, phospho-PKC and cryopyrin expression were reduced in the presence of Peps A and PMX205 (Fig. 4E). The induction of C5a-C5aR1 axis accumulates intracellular reactive oxygen species (ROS) [28, 30]. We observed accumulation of intracellular ROS in active THP-1 cells following exposure with spike CM or exogenous C5a as a positive control (Fig. 2 F). Our results indicated that alarmin mediated phagocytosis introduces a C5a-C5aR1 autocrine signal in activated THP-1 cells. Further, we measured the level of complement factor C5a by ELISA from the serum samples of uninfected healthy volunteers, severe COVID-19 patients, and recovered post-COVID-19 patients. A significantly higher C5a level was observed only from severe COVID-19 patient sera (Fig. 4G).

### Autocrine signal from C5a initiates extracellular trap formation by THP-1 cells

**Fig. 5:**
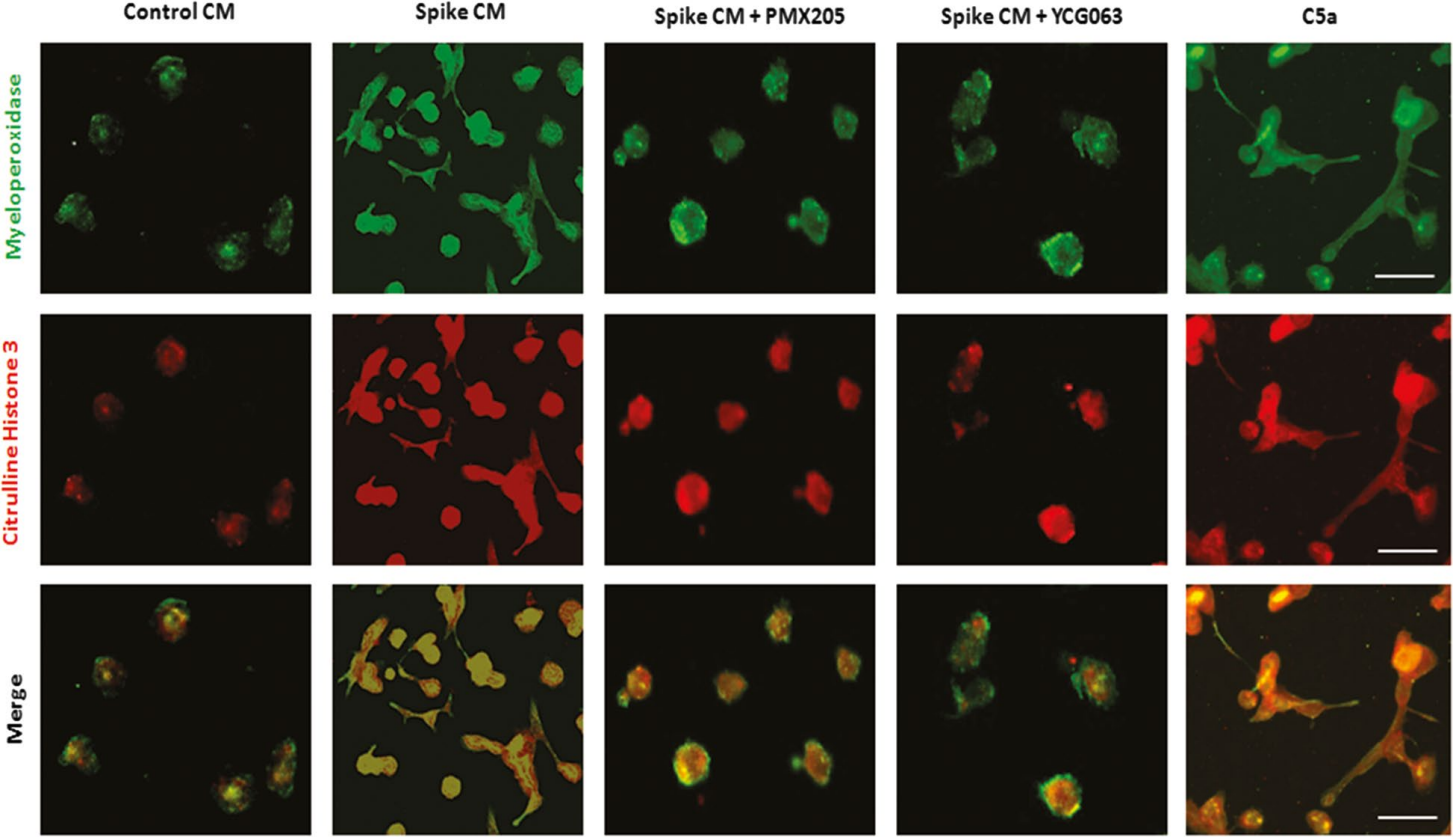
Role of C5a-C5aR1 axis in monocyte mediated extracellular trap formation. Monocyte extracellular trap formation was observed by fluorescence double staining with myeloperoxidase (green) and citrulline histone 3 (red) of THP-1 cells as markers after exposure to CM from spike expressing cells incubated with or without C5a inhibitor PMX205 and ROS inhibitor YCG063 for five days. Exogenous addition of C5a in cultures used as positive controls are shown on the right. Scale bars are 10 micrometers

Recent studies have demonstrated monocytes can produce extracellular traps in response to pathogen related innate immune response, where accumulation of intracellular ROS was required for extracellular trap formation. ROS induces histone degradation, membrane disintegration, DNA decondensation; followed by upregulation of elastase, myeloperoxidase, citrulline histone 3 protein; leading to decorate extracellular chromatin network complex with these proteins [31–34]. Complement activation or specifically C5a generation primes the extracellular trap formation [35]. Immunofluorescence staining of myeloperoxidase and citrulline histone 3, suggested extracellular trap formation by THP-1 monocytes exposed to spike CM and exogenous C5a as a positive control, whereas treatment with C5aR1 specific inhibitor PMX205 and ROS inhibitor YCG063 blocked extracellular trap formation (Fig. 5). Our results suggested that SARS-CoV-2 spike protein indirectly stimulates monocyte mediated extracellular trap formation.

### Monocyte extracellular trap initiates prothrombogenic stimulus

**Fig. 6:**
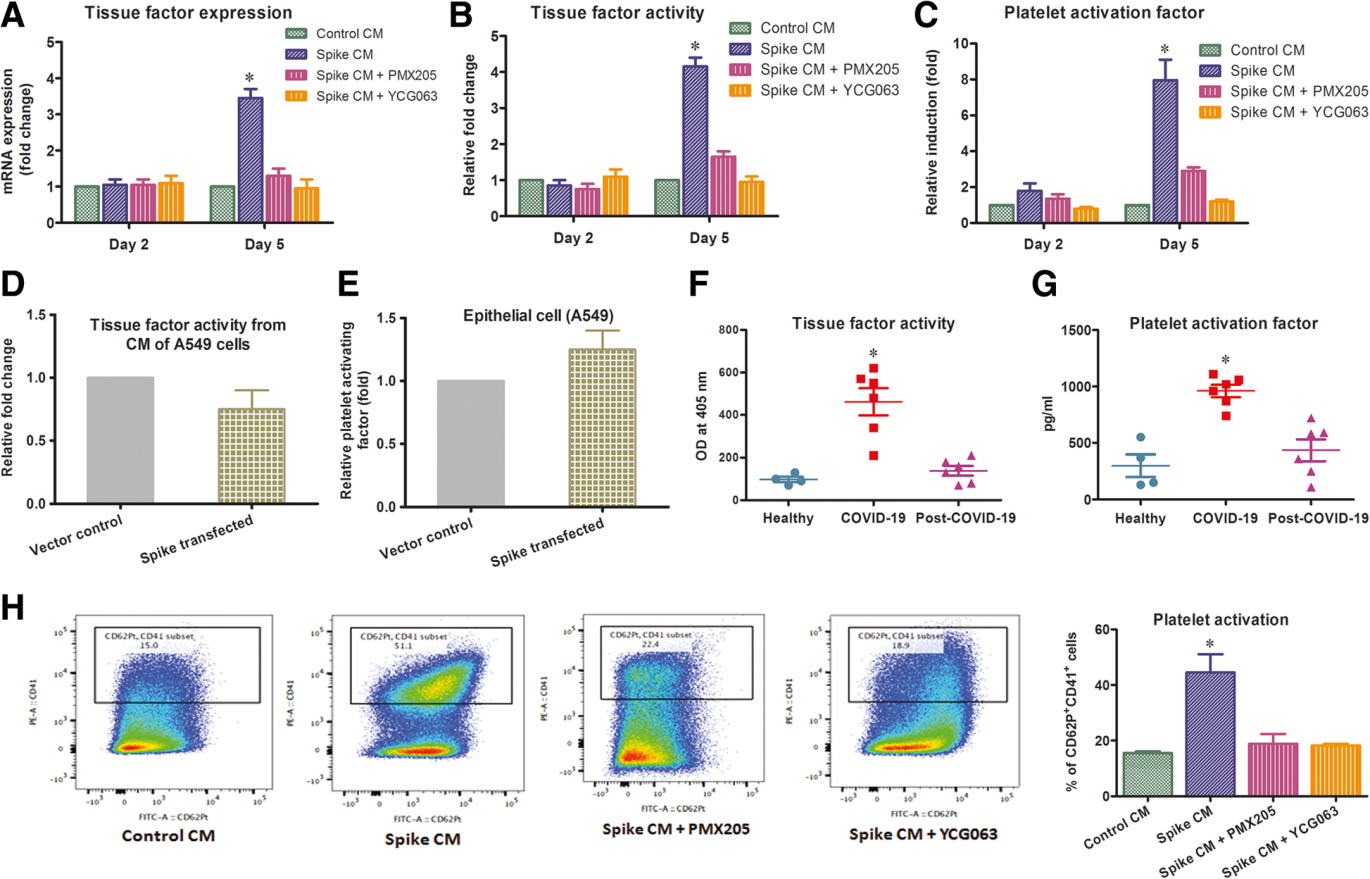
Prothrombogenic condition generation following monocyte extracellular trap formation.** A** mRNA expression of tissue factor was analyzed by qRT-PCR using total THP-1 RNA from cells exposed to CM from spike expressing cells with or without PMX205 and YCG063 treatment for two or five days. **B** Tissue factor activity was examined by colorimetric assay from culture supernatant of THP-1 cells exposed to CM from spike expressing cells in the presence or absence of PMX205 or YCG063 for two or five days. **C** Relative induction of platelet activation factor was analyzed by ELISA from the culture supernatant of THP-1 cells exposed to CM from spike expressing cells in the presence or absence of PMX205 or YCG063 for two or five days. **D** Relative tissue factor activity and (**E**) secretion of platelet activation factor level in the CM of A549 cells transfected with SARS-CoV-2 spike gene or empty vector is shown. **F** Tissue factor activity and (**G**) platelet activation factor were analyzed by ELISA from serum samples of uninfected healthy volunteers (*n* = 4), severe COVID-19 patients (*n* = 6) and recovered post-COVID-19 patients (*n* = 6). **H** Platelet activation was analyzed by flow cytometry using healthy volunteers cocultured for one hour with previously CM activated from spike expressing THP-1 cells on day five. The percentage of cells expressing platelet activation markers (CD62P and CD41) in different conditions are shown by histogram and bar diagram. The results are presented as mean ± standard deviation and ‘*’ represents analyses of data only showing statistical significance *p* < 0.05

Immune cells like neutrophils, monocytes, macrophages are the first to arrive at the site of endothelial damage even before platelets to initiate the thrombosis process. The extracellular trap governs by these immune cells stimulate thrombosis in both platelet dependent and independent ways. These immune cells upregulate tissue factor expression by extracellular trap formation to initiate thrombosis. Alternatively, extracellular trap release phosphatidylserine like platelet activation factors, DNA and histones to activate and aggregate platelets for thrombus formation [36–38]. In this study, we measured mRNA expression of tissue factor by qRT-PCR at different time intervals in THP-1 cells incubated with spike CM. On the day four when the THP-1 extracellular trap started to visualize, tissue factor mRNA was upregulated in the spike CM treated THP-1 and treatment of PMX205 or YCG063 prevented this upregulation (Fig. 6 A). The activity of tissue factor was assessed by colorimetric conversion of Factor-Xa from THP-1 cells following earlier reports [39–43]. Our results showed highest tissue factor activity of spike CM treated cells in day five not in day two (Fig. 6B). Additionally, we also determined the level of platelet activation factor from the culture supernatant of THP-1 cells by ELISA method. The platelet activation factor level was elevated on day five in spike CM exposed cells and treatment of PMX205 or YCG063 inhibited the elevation (Fig. 6 C). We did not get a considerable tissue factor activity and platelet activation factor in the culture media of SARS-CoV-2 expressing A549 cells (Fig. 6D and E). Tissue factor activity and concentration of platelet activating factor were assessed similarly in serum samples of uninfected healthy volunteers, severe COVID-19 patients and COVID-19 recovered patients. Both the tissue factor activity and the concentration of platelet activating factor were significantly higher in severe COVID-19 patient sera (Fig. 6 F and G). The marker CD62P or p-selectin and CD41 or glycoprotein IIb/IIIa integrin are expressed on the cell surface of activated platelets [44]. Platelet activation was measured by flow cytometry using CD62P and CD41 double positive stained cells as described earlier [45–49]. Healthy volunteer platelets cocultured for one hour either with extracellular trap forming THP-1 exposed with spike CM or with control CM. Highest platelet activation in the condition were detected where monocyte extracellular network was already established by spike CM treatment (Fig. 6 H). Thus, our results demonstrated that SARS-CoV-2 spike protein generates a paracrine effect on THP-1 monocytes to initiate prothrombogenic stimulus with the formation of extracellular trap.

## Discussion

SARS-CoV-2 infection normally localizes in pulmonary epithelial and ciliated airway cells [5–8, 50, 51]. The infected cells successfully potentiate monocyte activation with the advancement of a hyper-inflammatory condition which subsequently leads to acute respiratory distress syndrome (ARDS), because of severe COVID-19 disease [11, 12, 52]. Here, we used SARS-CoV-2 spike protein expressing A549 pulmonary epithelial cells and human monocytic cell line THP-1 as an in vitro model representing of virus infected cell and systemic monocyte communication in facilitating disease manifestation. Our results highlight SARS-CoV-2 spike protein expressing epithelial cells modulate monocyte response to generate a prothrombogenic stimulus in acute COVID-19 patients. Our previous studies demonstrated that SARS-CoV-2 virus infected or virus spike transfected epithelial cells exhibit senescence and release of inflammatory molecules including alarmins and behave as chemoattractant for leukocytes [15, 18]. Recent clinical studies reported SARS-CoV-2 infected pneumocytes exhibit senescence phenotype releasing IL-1α [53–55]. Removal of virus mediated damage or senescent cells for maintaining tissue homeostasis, monocytes or macrophages pronounce phagocytosis by sensitizing alarmin molecules through MyD88 activation [56]. Generally, these inflammatory molecules are responded by TLR-MyD88 related pathway of activated monocytes for secreting pro-inflammatory cytokines as well as phagocytosis [20]. Our current study revealed that the THP-1 monocytes exposed with alarmins available in the CM of SARS-CoV-2 spike expressing epithelial cells secrete primarily pro-inflammatory cytokines TNF-α, IL-6, IL-8 and IL-1β. This cytokine signature also correlated with the serum of severe COVID-19 patients used in this study as observed earlier [57]. Alarmin sensed phagocytosis of THP-1 monocyte is depicted for the first time in our findings and correlated with COVID-19 patients-based data [57–59]. Our study also suggests the use of MyD88 inhibitor for suppression of hyperactive condition of monocytes and may be benefited in clinical situation.

The complement activation product C5a can generate from phagocytic cells [26, 27]. We observed phagocytic THP-1 cells release C5a which governs an autocrine signal through interaction with its receptor C5aR1. Presence of aspartic protease inhibitor Pepstatin A inhibited this autocrine signal. High C5a level and C5aR1 expression were observed in blood and pulmonary myeloid cells in proportion to the severity of COVID-19 disease [3, 4]. Our results from patient sera indicated elevated C5a level in severe COVID-19 cases. C5a is known as a stimulus of NETs formation and has been observed in severe COVID-19 [38, 60, 61]. Signal from C5a-C5aR1 axis activates PKC and NLRP3 inflammasome for increasing intracellular ROS and histone citrullination that ultimately forms extracellular traps [30, 35]. Our study suggested SARS-CoV-2 spike protein indirectly stimulates monocyte mediated extracellular trap formation; and treatment with C5aR1 specific inhibitor PMX205 or ROS inhibitor YCG063 blocked the trap formation.

**Fig. 7:**
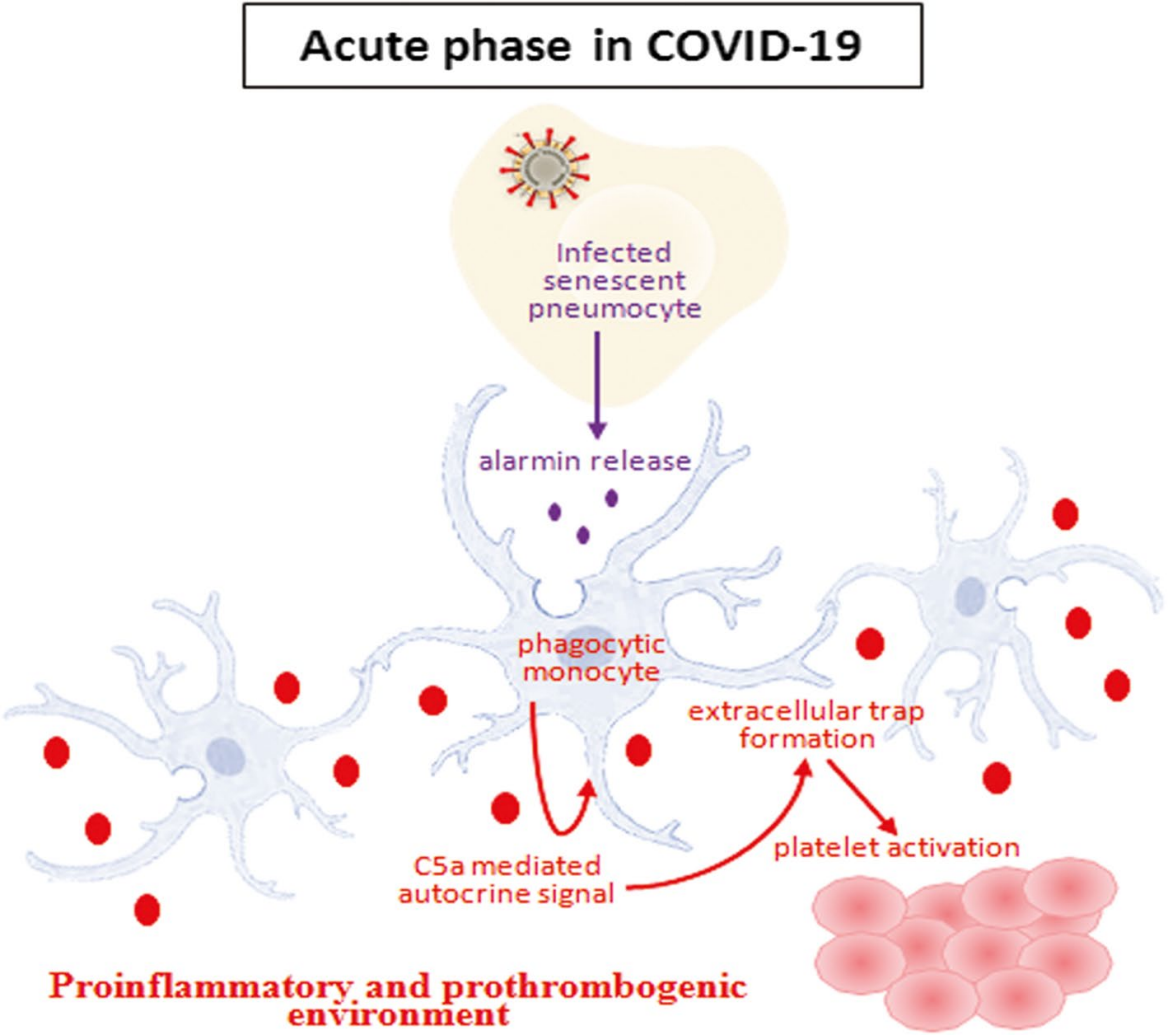
A schematic presentation of acute phase COVID-19 where virus infected cells potentiate a bystander effect on systemic monocytes for generation of thrombo-inflammatory environment

Platelet activation and aggregation are prothrombotic stimulus and involves in thrombus formation. Importantly, platelet-rich thrombosis is observed at multiple organs in post-COVID-19 autopsy specimens implying thrombosis contributes to COVID-19 related mortality with organ damage [38, 62]. Our results suggested that spike CM activated THP-1 following extracellular trap triggers tissue factor activity along with platelet activating factor production leading to platelet activation (Fig. 7). We also found increased tissue factor activity and elevated platelet activation factor in severe COVID-19 patient sera used in our study. Upregulated tissue factor expression, platelet activation and platelet-monocyte aggregate formation were also observed in severe COVID-19 patients [46]. Interestingly in real scenario, there are already some reports of thrombocytopenia cases after COVID-19 vaccination with spike protein [63–65]. However, we observed interruption of C5a-C5aR1 axis and ROS scavenger impair prothrombogenic condition. A preliminary cohort study with anti-C5aR1 antibody Avdoralimab in COVID-19 patients with severe pneumonia also showed inhibition of thrombosis and prevention of organ damage [3].

## Conclusion

COVID-19 research to date has primarily focused on virus infected cells, endothelial cells, and lymphocytes, but the other cells involved in systemic circulation like monocytes, platelets, or neutrophils are less emphasized. An important strength of our study is the highlighted alterations to monocyte phenotypes which presumably imitate the acute phase of COVID-19. Our present results will help to understand the pathogenesis of COVID-19 in a better way which may give rise to advancement of therapeutic modalities of critical COVID-19 patients specifically aiming on mononuclear cells.

## Data Availability

The data set used and analyzed during the study are available from the corresponding upon reasonable request.
